# Electrohydrodynamic Jet Printing: Introductory Concepts and Considerations

**DOI:** 10.1002/smsc.202100073

**Published:** 2021-11-07

**Authors:** Nhlakanipho Mkhize, Harish Bhaskaran

**Affiliations:** ^1^ Department of Materials University of Oxford Parks Road Oxford OX1 3PH UK

**Keywords:** electrohydrodynamic jet printing, inkjet printing nozzles, ink properties, process parameters for jet printing, substrate properties for inkjet printing

## Abstract

Electrohydrodynamic (EHD) jet printing is an emerging technique in the field of additive manufacturing. Due to its versatility in the inks it can print, and most importantly, the printing resolution it can achieve, it is rapidly gaining favor for application in the manufacture of electronic devices, sensors, and displays among others. Although it is an affordable and accessible manufacturing process, it does require excellent operational understanding to achieve high resolution printing of up to 50 nm as reported in literature. In this review, three main aspects are considered, namely, the ink properties, the printer system itself (including design, nozzle dimensions, applied potential, and others), and the substrate onto which printing is being carried out. Knowing how all these factors can be manipulated and brought together allows the users of EHD printing to achieve extraordinary resolution and consistent results. The review is concluded with a brief discussion on where one can see the potential for development in this field of research.

## Graphical abstract

Electrohydrodynamic (EHD) jet printing is a high‐resolution additive manufacturing technique which has gained significant attention in recent years. It is an inexpensive tool which has found use in a variety of manufacturing areas. Whilst accessible, EHD printing requires good understanding of factors such as the ink properties and system parameters. This review offers insights into its successful application.

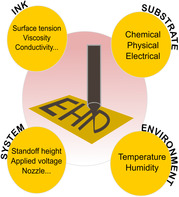

## 1 Introduction

### 1.1 Definitions

Electrohydrodynamic (EHD) jet printing is a noncontact printing technique, which has gained much attention in recent years.^[^ ^1^, ^2^, ^3^ ^]^ It works by applying an electric field to induce ink ejection from a conductive nozzle onto a substrate. EHD jet printing is a specific application of the well‐studied electrohydrodynamic atomization (EHDA).^[^ ^4^ ^]^ **Table** **1** shows the different phenomena which fall under this umbrella term.^[^ ^5^, ^6^, ^7^ ^]^ For the purposes of this work, we will refer to near‐field (less than 1 mm) cone‐jet printing as EHD.

**Table 1:** EHD atomization modes as summarized by Jaworek and Krupa.^[^ ^6^ ^]^

EHDA category	Subcategory	Description
Dripping mode	Dripping	Large drops (≈ nozzle diameter) pinch off when the electric field is switched on.
		Characteristic of flow rates faster than jet formation
	Microdripping	Small droplets pinch off from meniscus
		Characteristic of flow rates slower than jet formation
	Spindle	A large spindle shaped fragment is pinched off before a jet can occur
		Characteristic of an extremely high electric field
		Flow rate is equivalent to jetting rate
Jet mode	Cone jet	A stable cone and jet form
		Less space charge results in less interference allowing jet to remain along the central axis
	Multijet	A stable cone forms, but multiple jets arise
		Likely due to fast flow rate, or excessive applied potential
	Precession jet	A steady cone and jet are formed, but precess about the nozzle circumference
		Caused by electrostatic force build up due to sprayed droplets (space charge)
	Oscillating jet	Flow rate sufficient to create a steady jet, however off the central axis
		Caused by excessive charge build‐up

**Figure 1:**
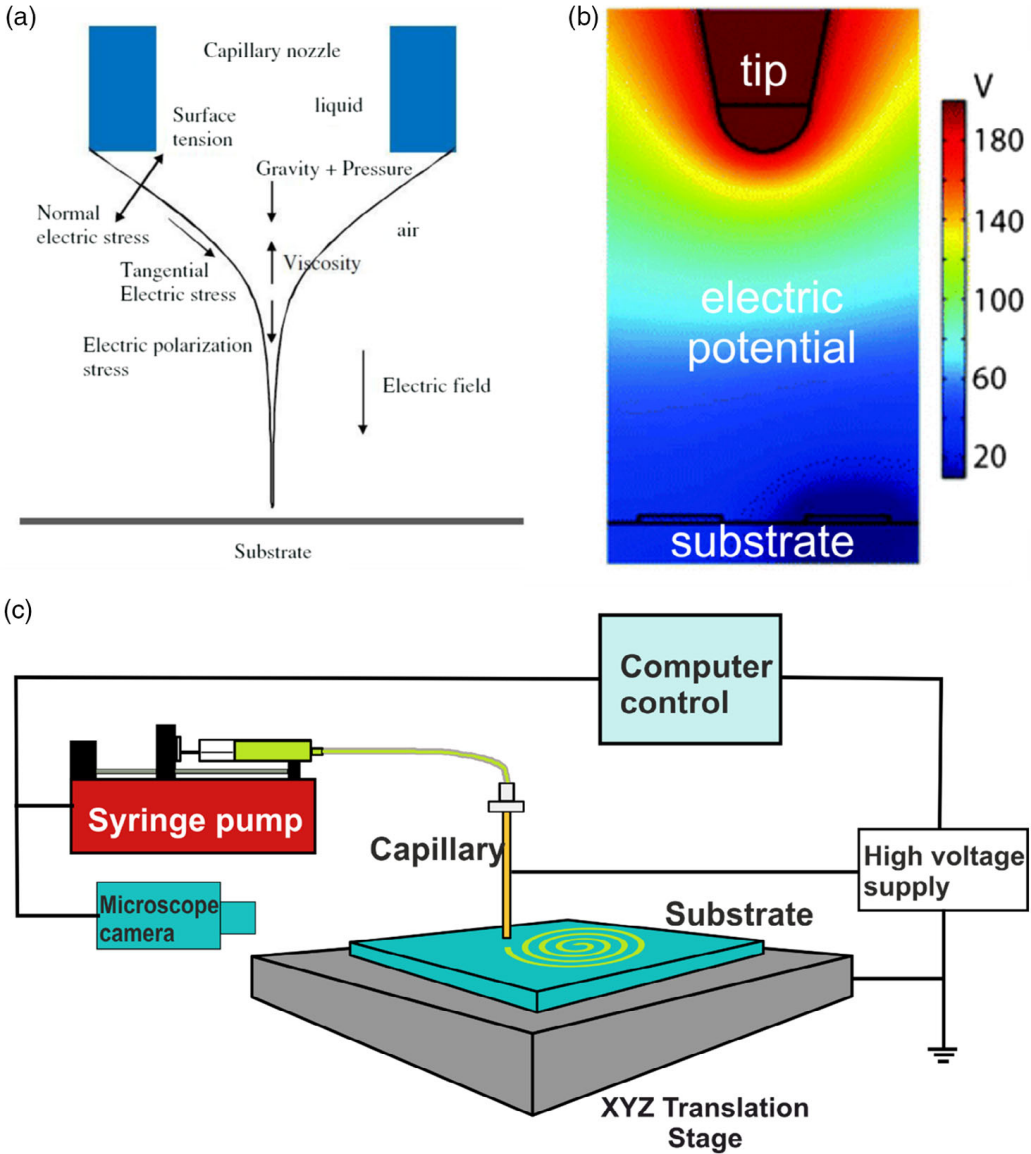
Physics of electrohydrodynamic jet printing. (a) Summary of forces acting at capillary tip during EHD printing using the cone‐jet mode. Reproduced with permission.^[^ ^8^ ^]^ Copyright 2010, IOP Publishing. (b) The model shows the spatial distribution of the electric potential during EHD operation around the tip, with the scale bar indicating field strength. Adapted with permission.^[^ ^9^ ^]^ Copyright 2016, Royal Society of Chemistry. (c) Simple schematic of the components required to build an EHD printer.

For these EHDA methods, the electrostatic force resulting from the normal component of the electric field is sufficient to overcome the surface tension of the ink, as shown in **Figure** **1**a.^[^ ^8^ ^]^ Figure 1b shows how the magnitude of the electric potential around a nozzle looks like in space.^[^ ^9^ ^]^ Figure 1c shows a simple schematic of the setup required to carry out EHD printing. A conductive capillary is hooked up either to a syringe pump or air supply to supply the ink. The high voltage source provides the electric force required to induce the printing. The translation stage holds the substrate being printed on and controls how the deposition pattern is formed, usually a predetermined design loaded into control software. This stage can also translate in the *z* direction, and this modulates the strength of the electric field (for constant potential). The microscope camera allows for visualization of the printing process.

An ink in a capillary, as shown in Figure 1a, will not flow until a force is applied which will overcome the surface tension and capillary forces by which it is held stationary. The only forces pushing down on it are a weak gravitational force and any pressure which exists within the system (due to the syringe pump or applied air pressure). When an electric potential is applied to the system, charge will migrate to the meniscus surface of the ink, depending on whether it has mobile charge carriers or not. The conductivity of the ink depends on the number of charge carriers per unit volume and their drift velocity.^[^ ^10^ ^]^ When sufficient charge has built up on the ink interface, an electrostatic potential is created between the meniscus and the grounded substrate. The electrical stress now present has a normal and tangential component, as the inks are generally not perfect conductors.^[^ ^11^ ^]^ The normal stress destabilizes the meniscus and the tangential component supports the formation of the meniscus into a cone (termed a Taylor cone^[^ ^12^ ^]^) which results in jetting. This was modeled numerically by Rahmat et al. and found to be consistent. The electric forces pull the ink toward the deposition substrate.^[^ ^13^ ^]^ This works best for liquids which are sufficiently dielectric, meaning that they can sustain the electric field.^[^ ^14^ ^]^ Where the dielectric constant is too low, errant satellite droplets are possible.^[^ ^15^ ^]^ Early work by Hayati et al. on the mechanism of stable jet formation in EHDA demonstrates visually the effect of the electrical stresses on a fluid by showing the circulation patterns which can arise.^[^ ^16^ ^]^

**Figure 2:**
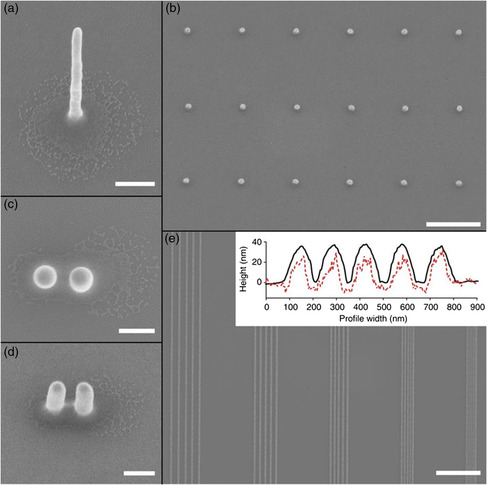
a) Gold nanopillar of diameter ~50 nm and aspect ratio of ~17 (scale bar, 200 mm). b) Top and c) side views of nanopillars printed subsequently at 200 nm center‐to‐center distance (scale bar, 200 nm). d) Dots of 80 nm wide printed into a 1 μm lattice constant array (1 μm scale bar). e) Printed tracks with pitch sizes of 250, 200, 150, 100, and 75 nm (scale bar, 2 μm). The inset shows atomic force microscopy (AFM) (full black lines) and scanning electron microscopy (SEM) (red dashed lines) profiles of 150 nm pitch size. The height of AFM profiles is given in nanometers. The SEM profiles are in arbitrary units. Tracks have reproducible heights of ~40 nm and are well separated. Reproduced with permission.^[^ ^20^ ^]^ Copyright 2012, Springer Nature.

The advantage of EHD printing comes from the formation of a characteristic Taylor cone (first described by Sir Geoffrey Taylor). The jet will be of smaller spatial dimensions than the nozzle used if the correct conditions are applied, unlike what is observed with conventional inkjet printing.^[^ ^17^ ^]^ Taylor found that for an ideal conducting liquid, the angle at the cone apex is 2*θ*_T_ = 98.6°.^[^ ^18^ ^]^ This size reduction in jet diameter is especially important in pursuits such as nanofabrication, where the size limits of devices and structures are currently restricted with existing extrusion technologies, such as inkjet printing.^[^ ^1^, ^19^ ^]^ With EHD, high aspect ratio printing of up to 50 nm lateral resolution is shown (**Figure** **2**a‐e).^[^ ^20^ ^]^ The versatility of the process is further enhanced by the wide variety of materials which can be deposited, in any configuration, without the need for set templates or masks.^[^ ^1^, ^21^ ^]^ It is a true additive manufacturing technique as only the material required is deposited, thus eliminating waste.^[^ ^22^ ^]^ This is one of the reasons why the technique is touted as being one of the most economical manufacturing techniques available. The mathematics of the stable jet mode is beyond the scope of this work, but can be found in seminal work done by Hohman et al.^[^ ^23^ ^]^ Other jet modes have been reported by Jaworek and Krupa.^[^ ^6^ ^]^ The advantages and disadvantages of EHD compared with other direct writing techniques are shown in **Table** **2**.

**Table 2:** Additive manufacturing techniques. A comparison of the advantages and disadvantages of some commonly used additive manufacturing processes on the microscale and nanoscale

Technique	Advantages	Disadvantages	References
Inkjet printing	Noncontact patterning technique thus safe to use for multilayer processes	Resolution limited by nozzle diameter	[159]
	Variety of materials processable	Unable to process materials with high viscosity	
	Does not require vacuum	Prone to nozzle clogging	
	Requires no mask or template	Mainly used for planar patterning	
	High speed	Poor drying leads to inhomogeneous films via the “coffee ring effect”	
	Useful for micro‐ and macroscale printing		
	Widely commercialized		
	Highly reproducible		
	Compatible with roll‐to‐roll processes		
Microcontact printing	Remarkably high resolution	Contact mode patterning	[160, 161, 162, 163, 164]
	Rapid fabrication process	Requires fabrication of template stamp using elastomer.	
	Does not require vacuum or high temperatures	Limited to planar 2D structures	
	Allows for many materials to be patterned	New stamps required for different patterns	
	Wide applicability		
	Compatible with flexible substrates		
Dip‐pen lithography	Exceptional nanoscale resolution (15 nm)	Large printing tip arrays require complex fabrication	[32, 165, 166, 167, 168]
	Scalable with multitip arrays	Mechanically fragile tips	
	No substrate modification required to achieve high resolution	Expensive equipment required	
	Able to deposit sensitive biological matter	Limited material patterning	
	Does not require vacuum conditions		
	Unlimited pattern design		
Gravure printing	High throughput	Suitable only for planar printing	[169, 170]
	High speed (1 ms^−1^)	Rigid substrates difficult to process	
	Wide range of ink viscosity processable (10–200 cP)	High initial setup cost	
	Sub‐10 μm resolution		
EHD jet printing	Resolution not limited by nozzle diameter	Prone to nozzle clogging	[1, 2, 171, 172, 173]
	Scalable (nano‐ to microscale printing)	Glass tips are easily broken	
	3D structures can be created	Only solutions/suspensions processable	
	Wide variety of materials can be processed	Challenging to print on insulating substrates	
	Rigid and flexible substrate compliant	Substrate damage possible if excess potential applied	
	Low setup cost	Printing varies strongly with varying field strength	
	Highly tunable process	Crosstalk probable in multi‐tip arrangements	
	Process hybridization possible		
	Multiple tips can be used in tandem		

### 1.2 Scope of Review

To fully make the best of the benefits of EHD printing technology, the interplay between properties of materials used and system parameters must be understood.^[^ ^7^, ^24^, ^25^ ^]^ The interaction of the electric field with the chosen ink relies heavily on the system's parameters including applied potential, stand‐off height, nozzle diameter, and operating environment (e.g., humidity, temperature, etc.).^[^ ^26^ ^]^ In addition, there are properties of the ink that require due consideration, namely surface tension, viscosity, conductivity, and fluid nature. In the following sections, we look at each of these named variables in turn and relate them to the overall EHD process.^[^ ^27^, ^28^ ^]^ Several key studies looking at the interplay of only some of these variables have been reported.^[^ ^28^, ^29^, ^30^ ^]^ Lee et al. and Choi et al. have done significant work in describing the majority of these parameters.^[^ ^28^, ^31^ ^]^

The aim of this work is to enhance understanding of the EHD process and highlight the intricacies involved to achieve the best possible result and resolution, based on existing methodologies, as well as those which we describe here. For applications of EHD printing, and current progress, several excellent reviews exist within the larger framework of additive manufacturing,^[^ ^1^, ^32^, ^33^, ^34^, ^35^ ^]^ biological studies,^[^ ^36^, ^37^, ^38^, ^39^ ^]^ flexible sensors,^[^ ^40^ ^]^ and electronics.^[^ ^41^ ^]^ The references cited in this work are not exhaustive, and were chosen due to their instructive or illustrative nature.

## 2 Ink Considerations for EHD Printing

Inks are at the core of EHD printing. Their properties, and intended functionality, determine the ideal printer settings. The inks available can be categorized as homogenous solutions (pure solvents or solubilized material), suspensions (such as colloids of quantum dots, nanoparticles, insoluble material), polymers, melts (such as molten metal, wax, etc.), and biomolecular inks (DNA, proteins, and bacteria). **Table** **3** shows just some of these many inks and their applications. As an essential component, understanding how the ink properties affect EHD printing is of the utmost importance. In this section, we examine a few of the properties and outline their implications.

**Table 3:** Selected functional inks used in EHD printing

Material	Function/application	Resolution	References
Polyaniline	Chemiresistive sensor	≈200 μm	[79]
Polycaprolactone	Polymer scaffold	50–80 μm	[76]
	Tissue regeneration scaffold	193 nm	[174]
Zinc‐tin‐oxide	Oxide thin‐film transistor	200 μm	[69]
Polydimethylsiloxane	Bio‐scaffolds	≈50 μm	[175]
Quantum dot suspension	Optical Display	Spray	[176]
Ag nanoparticles	Electrodes	≈100 μm	[177]
Ag nanoparticles	Flexible strain sensor	50 ± 5 μm	[152]
Poly‐hydroxymethylglycolide‐*co*‐ε‐caprolactone	Cardiac tissue scaffolds	4–7 μm	[178]
BP212 photoresist	Lithography	9 μm	[150]
PEDOT:PSS‐PEO	Flexible microdevices	30 μm	[127]
Perovskite IL ink	Flexible perovskite photodetector	1 μm	[101]
3‐Aminopropyl triethoxysilane	Nanoparticle assembly	300 nm	[129]
Wax (melt)	3D microstructures	< 10 μm	[24]
Protein inks	Cell attachment	5 μm	[96]
PZT nanostructures	Piezoelectric	40 nm	[115]
Colloidal crystals	Structural color	10.2 μm	[179]
Glucose oxidase	Glucose sensor	≈5 μm	[180]
Graphene oxide	Energy storage device	< 100 μm	[181]
Ag nanoparticle‐PEO blend	Flexible transparent electrode	8 μm	[182]
Optical resin Ag paste	Microlens arrays Heater electrodes	≈100 μm ≈50 μm	[183]
Bacterial cellulose	Bioscaffolds	≈100 μm	[155]
Alginate Bioladen collagen/CaCl_2_	3D prevascularized cardiac constructs	440 μm 224 μm	[156]

### 2.1 Surface Tension

A free droplet, unencumbered by electrostatic, aerodynamic, or gravitational forces will always assume a spherical shape. This is because of a sphere costing the least energy to form. This minimization of energy (and areal coverage) is because of the surface tension (*γ*) of the molecules. This property is a consequence of the fact that molecules at the free surface (liquid–air interface) have higher energy than within the bulk.^[^ ^42^ ^]^ When we consider the droplet on a solid surface, there exists a force which is a result of the surface tension. This force acts in the plane of the free surface, perpendicular to a free edge in that surface. The force (*F*) is proportional to the length (*L*) of the edge.

(1) F=γL

Surface tension is an important parameter to consider for the inks used in EHD printing. The electrical force actuated needs to be able to overcome the surface tensile force with which the meniscus is held to the capillary.^[^ ^43^ ^]^ It has been demonstrated by He et al. that if the surface tension is too low, the ink will form satellite droplets before a stable jet can be formed, due to a longer pinch off time (the time it takes for a droplet to detach from the jet).^[^ ^44^ ^]^ Studies also show that if the surface tension is too high, the electrostatic force applied will not be sufficient to cause the jetting of the material,^[^ ^45^ ^]^ and only meniscus pulsing will be observed, resulting in no printing. Several articles make reference to a specific range of surface tensions which are considered useful or optimal for EHD printing.^[^ ^28^, ^46^ ^]^ “Islands of stability” have been demonstrated, considering other properties such as conductivity and viscosity, which we discuss shortly.

To understand the influence of the surface tension on EHD, let us first look at a very simplified mathematical treatment. A basic condition for jetting to occur is that the electrostatic force be sufficient to overcome the surface tension (Equation (2))

(2) $$F_{\text{surface tension}}=F_{\text{electrostatic}}$$

If we treat the force between the capillary and the ground plane as being capacitive and the surface tensile force as being the product of the surface tension and the distance over which the force is applied, we obtain Equation (3)

(3) $$\gamma L=\frac{\varepsilon_{o}AV^{2}}{2d^{2}}$$

where *γ* is the surface tension of the ink, in N/m, *L* is the distance over which surface tension (in m) applies (in this case circumference of the pipette), *A* is the area over which the electric field is experienced, *V* is the applied potential, and *d* is the stand‐off height. Rearranging for *V*, we obtain the following (Equation (4)).

(4) $$V=\sqrt{\frac{2\gamma Ld^{2}}{\varepsilon_{0}A}}$$

**Figure 3:**
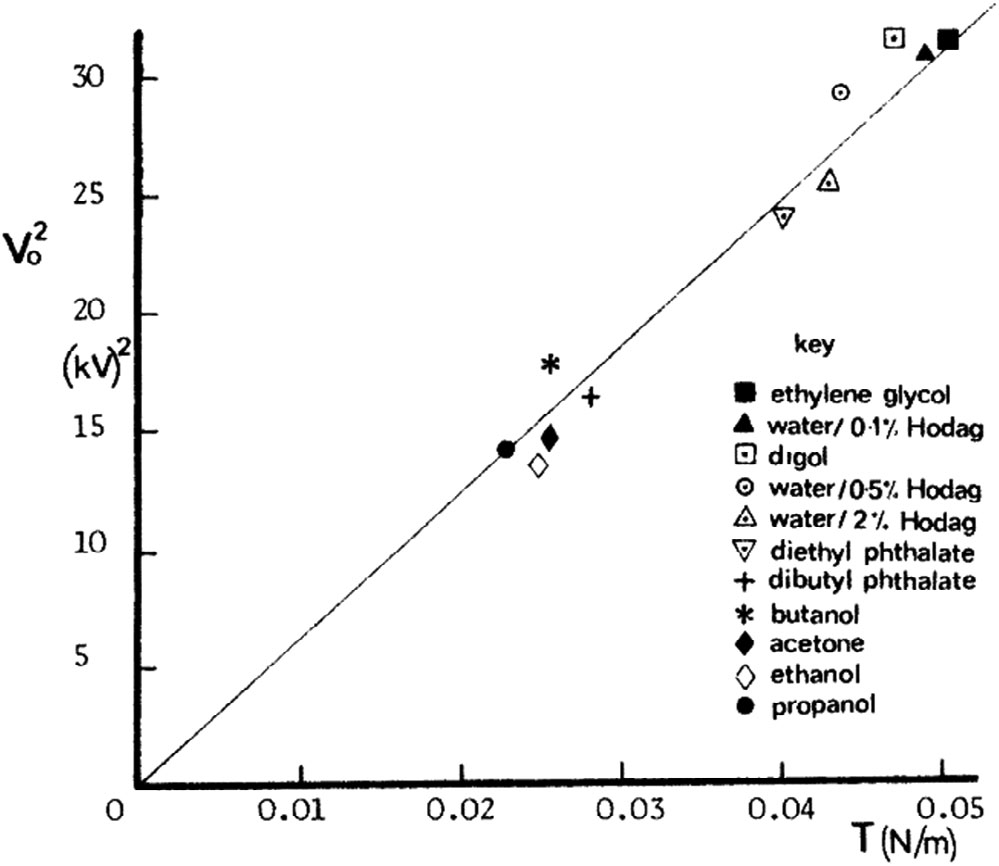
Effect of surface tension on applied voltage. Reproduced with permission.^[^ ^47^ ^]^ Copyright 1986, IEEE.

From this, we see that *V* ∝ *γ* ^0.5^. The applied potential is directly proportional to the square root of the surface tension. Smith plotted this relationship for a series of common solvents and found a linear trend (**Figure** **3**).^[^ ^47^ ^]^ The higher surface tension of some solvents is a result of the strong intermolecular forces, such has hydrogen bonds. This relationship is notable, as it means that the applied voltage does not increase rapidly over a range of surface tensions, which a single material could exhibit—especially under variable temperature conditions.

Using two different inks, Choi et al.^[^ ^31^ ^]^ derived more involved scaling laws that enable further understanding of the relationship between the electric field strength, the jet pulsing frequency, and diameter as the jet leaves the capillary tip. They carried out EHD experiments using several capillary sizes, and also compared existing literature. As a starting point, they describe the flow rate of the jet using the Poiseuille‐type flow rate equation (Equation (5)) first described by Chen et al.^[^ ^48^ ^]^

(5) $$Q\approx \frac{\pi d_{N}^{4}}{128\mu L}(\Delta P+\frac{1}{2}\varepsilon_{o}E-\frac{4\gamma }{d_{N}})$$

where *Q* is the flow rate, *d*_N_ is the diameter of the nozzle, *μ* is the viscosity of the ink, *L* is the nozzle length, Δ*P* is the change in pressure, *ε*_o_ is the permittivity of free space, *E* is the electric field strength, and *γ* is the surface tension on the meniscus. Approximating the electric field *E* is done using a model of a semi‐infinite wire perpendicular to an infinite planar electrode (Equation (6))

(6) $$E=4V_{0}/[d_{N}\ln(\frac{8H}{d_{N}})]$$

where *V*_*0*_ is the applied potential difference and *H* is the distance between the tip and substrate. We shall refer to this distance from now on as the stand‐off height. Through a series of derivations, which are not part of the scope of this review, Choi presents two scaling laws which can give the relative magnitudes of important parameters mentioned earlier. The jet diameter scales according to Equation (7)

(7) $$d\propto \sqrt{\frac{\gamma }{\varepsilon_{0}}}\frac{\sqrt{d_{N}}}{E}$$

which means for an increase in electric field, there is a linear decrease in jetting diameter. The second scaling law describes the relation between the jet pulsing frequency and the *E* field, and is stated as Equation (8)

(8) f∝(εo3ρ2γ)14E32dN34

where *ρ* is the density of the ink used. Having described these laws, they found satisfactory agreement with the experiments they carried out. Understanding these laws, and the deviations which occur from them (for different pressures or very low surface tension systems), is beneficial for the design of EHD systems. We note here that these are not the only scaling laws which have been developed, nor are they a completely rigorous description of EHD printing. They are merely a guideline which has been accepted by many researchers working in the field.

It is also important to note that surface tension is dependent on temperature.^[^ ^49^ ^]^ Rowlinson and Widom state that the surface tension of a liquid in equilibrium with its own vapor pressure decreases and becomes zero at the critical point as temperature is increased.^[^ ^50^ ^]^ However, this is highly unlikely to happen in EHD printing because of the tiny volumes used, it is noteworthy. Temperature effects are still nontrivial. The first major cause of temperature change would be the printing environment. It is ideal to keep conditions as constant as possible to ensure reasonable printing quality and reduce any inconsistencies.^[^ ^51^ ^]^ When an electric field is applied, temperature can be significantly changed (increased) if arcing is allowed to occur, or if the electrical conductivity of the ink is so low that jetting is not possible. This could lead to a point where the surface tension of the material being printed is altered, thus throwing off the balance between the surface and electrostatic force (see Figure 2a).^[^ ^52^ ^]^ The likely outcome of this would be a higher‐order jetting mode or excessive material being printed at any one time. This could be rectified with a feedback system which considers the changes in jetting rate. If the flow rate changes to a value lower than expected, the feedback system could effectively alter the voltage, thus rectifying the issue. In fact, such a system has been demonstrated by Kien Nguyen et al.^[^ ^53^ ^]^ Alternatively, a cooling system could be used to maintain the stability of the surface tension of the material during printing. A cooling system for instance can be used to control the substrate temperature to limit wetting. This method has been used to limit the “coffee ring effect” observed in inkjet printing.^[^ ^54^ ^]^ Short printing times could also mitigate the differential temperature issue.

Surface tension is one of the easiest properties to control in inks used in EHD printing. It has a significant effect on the magnitude of the potential required to induce printing. Surface tension also plays a large role in the consideration of the nozzle dimensions for a particular system. To mitigate any negative effects, it is essential to know what kind of surfactants can assist in controlling the surface tension to a desirable range.

### 2.2 Surfactants

In developing functional inks, the addition of surfactants can alter the surface tension, and subsequently, flow patterns considerably.^[^ ^2^, ^45^, ^55^, ^56^, ^57^ ^]^ Accurate characterization, or at least understanding, of the surface tension for all new ink blends before use is crucial to be able to predict the conditions they will require for effective printing. The addition of surfactants, which disturb the intermolecular forces and reduce the interfacial tension, can sometimes introduce new challenges to the processing of printed structures. Depending on the volume of surfactant added, the solvent evaporation temperature could be increased when trying to sinter or dry the printed feature. This is especially important where substrates on which the inks have been printed onto are not able to be heated very high (polymers, phase change materials, etc.).^[^ ^58^, ^59^, ^60^ ^]^ Excessive reduction of the surface tension must also be avoided, else over‐delivery of the ink is possible. Li et al. demonstrate a secondary serendipitous effect of surfactant in inks. They show that surfactants can boost the electrical properties of multiwall carbon nanotube inks by preventing aggregation which would reduce the number of viable electrical pathways through which charge could pass.^[^ ^61^ ^]^ Lanauze et al. study a droplet of liquid suspended in silicone oil in the presence of electric fields. Their liquid, squalane, exhibits deformation at a field strength of 7.6 kVcm^−1^. However, when sufficient surfactant is added, this drops down to 2.5 kV cm^−1^.^[^ ^62^ ^]^ However, they do not study the droplets within the framework of EHD printing, their results highlight the significance of surfactants on the EHD properties that inks can exhibit.

### 2.3 Colloids

**Figure 4:**
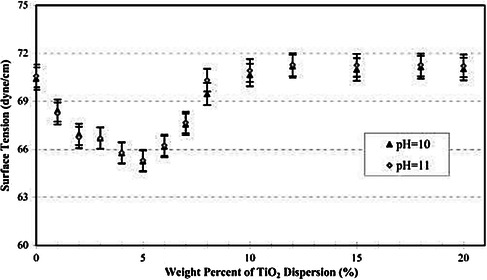
Surface tension responding to colloidal concentration. Plot of the surface tension versus weight percent of titania dispersions at pH 10 and pH 11. The plot shows that the surface tension of the dispersion decreases significantly from ≈0% to 5% and then increases above the surface tension of pure water at 10% and higher weight percentages. Reproduced with permission.^[^ ^63^ ^]^ Copyright 2003, American Chemical Society.

When considering colloidal suspensions used as inks, not only is surface tension important, but also the colloid concentration. A study by Dong and Johnson showed how the surface tension of an aqueous suspension decreases significantly as the concentration of titanium oxide increased, until a critical point was reached (**Figure** **4**). After this point, it is hypothesized that further increasing the concentration increases the surface tension because of interparticle capillary forces dominating, until a plateau is reached.^[^ ^63^ ^]^ This being the case, highly concentrated suspensions could lead to nozzle clogging because of particle aggregation, which increases the viscosity of the ink significantly.^[^ ^64^, ^65^ ^]^ Thus, dilute concentrations of colloidal suspensions tend to be used.^[^ ^66^, ^67^ ^]^

In an aforementioned study by Lanauze et al., the influence of a colloid within a droplet is also probed.^[^ ^62^ ^]^ By comparing the deformation behavior of a droplet of squalane suspended in silicone oil which contains carbon black particles, to a pure squalane droplet, they make two important observations. First, where the colloid is unstable, meaning that the particles are not homogenously distributed, flocculation occurs much more quickly upon the application of an electric field. In a stable colloidal solution (stabilized by sufficient surfactant), this does not occur as rapidly. Within an EHD paradigm, this means that the colloid ink stability is crucial to ensure homogenous printing and uniform structural deposition, at the appropriate field strength. The second observation made was that the presence of colloidal particles in a droplet containing surfactant sufficient to stabilize them electrostatically, did not have a different deformation shape than a droplet containing the surfactant alone. Looking at the implication of this observation on EHD printing, we can say that surface tension has a significant impact on Taylor cone formation than the conductivity contributed to the colloidal suspension by the particles.^[^ ^68^ ^]^

### 2.4 Solutions

In an ideal scenario, a completely solubilized ink should be used for printing, to avoid any issues of aggregation‐induced clogging. This is why reactive inks (mainly silver) have attracted much attention.^[^ ^69^, ^70^, ^71^ ^]^ The precursor is (usually) a metal salt or oxide completely dissolved in a solvent, with appropriate additives used to control the ink properties. It is printed and then exposed either to UV light or heating to achieve the desired metallic patterns. It is assumed also that solutions are Newtonian fluids. This means that the flow expected during printing should be linear and proportional to the applied forces. However, other types of inks exist.^[^ ^72^ ^]^

Viscoelastic fluids are mostly composed of high molecular weight materials such as polymers, and exhibit counter‐intuitive behavior. Due to their macromolecular nature, they exhibit elastic behavior at times, but also exhibit viscous behavior under certain conditions.^[^ ^73^ ^]^ Newtonian fluids behave more predictably. They respond linearly to the strain exerted on them. All inks used are viscoelastic to some extent. This makes characterizing them more elaborate. Where once there were known relationships between viscosity, flow rate, and the applied potential, viscoelastic liquids demand more complex treatment. For the purposes of this work, non‐Newtonian fluids will not be discussed at length. They are dealt with in work done by Feng.^[^ ^14^ ^]^

Inks which are emulsions of immiscible liquids or liquids and particles with different properties pose the threat of phase segregation when exposed to nonuniform electric fields (as are observed in EHD printing).^[^ ^74^ ^]^ Therefore, when using additives to alter solution properties, the quantities of the additives must be such that the possibility of phase segregation (especially for polymer solutions) is minimized. The problem that this separation could result in is the alteration of the desired properties of the printed structures. Where precipitation or aggregation is possible, a separation leads to a clogging of the printer nozzle.

Numerous studies exist where melts are used as inks. Han et al. demonstrated the patterning of a wax ink to form micropillar arrays, and high aspect ratio thin walls. Due to the rapid phase‐change the wax exhibits once printed (liquid to solid), 3D structures are easily obtained. These can be used as scaffolds for further fabrication.^[^ ^24^ ^]^ To achieve this, the authors had to modify their printer system to incorporate a heating element.^[^ ^75^ ^]^ The same team also presents the printing of Field's metal.^[^ ^19^ ^]^ To get the metal to print (despite its extremely high surface tension), the nozzle had to be heated to 380 °F (193 °C) before jetting could occur. On the opposite end of this temperature spectrum, Li et al. developed a printing system which incorporated a cryogenic workbench. This system was able to create surfaces with temperatures as low as −20 °C, which instantly solidified the polymer solution they were printing to create 3D structures with lateral resolution of between 50 and 80 μm.^[^ ^76^ ^]^

### 2.5 Conductivity

Where electrostatic actuation is required, the conductivity (*K*) of the ink used is of critical importance. The intrinsic charge mobility affects parameters such as Taylor cone formation time, charge relaxation time, and voltage required to cause flow (all these affected by viscosity too). Various time constants exist which describe these relationships, and are discussed presently.^[^ ^77^ ^]^

A vast amount of work done in the field of EHD printing has been for drop‐on‐demand (DOD)‐type work, however, more interesting is the application of this technique to continuous jet printing. Continuous jet printing is more favorable due to its scalability and commercial viability. Completely insulating solvents, such as hexane for example, will not allow for conventional jet printing. Zhang et al. studied a family of dielectric silicone oils which vary in viscosity but have similar surface tension, conductivity, and relative permittivity values.^[^ ^78^ ^]^ Due to their poor conductivity, these liquids are unable to exhibit cone‐jet behavior, but do demonstrate unstable transient jetting. Other solvents which are more conductive are better able to sustain EHD jet printing.^[^ ^47^, ^72^ ^]^

Various studies have been conducted in EHD with solvents which are considered more dielectric (*K* ≤ 10^−12^ S m^−1^) than conductive.^[^ ^28^ ^]^ By adding more charge carriers to solvents (by addition of salt solution or acid), one can increase the degree of conductivity, thus making the charge transfer process easier and facilitating EHD printing. The addition of dilute acid or salt solutions to inks has been demonstrated to increase the ionic concentration and thus improve the conductivity.^[^ ^47^, ^77^, ^79^, ^80^, ^81^ ^]^ The addition of acid however, can alter the physical nature of the solution, especially where polymers are involved. For instance, gelation/swelling of polyvinyl alcohol (which can be used as a surfactant) has been observed in the presence of hydrochloric acid.^[^ ^82^ ^]^ The precipitation of species is also a consequence of altering the pH of an ink by adding acid, as is demonstrated by Cao for polyaniline.^[^ ^83^ ^]^

#### 2.5.1 Parameters Influencing Conductivity

Depending on the type of ink used (whether polymer based, salt, colloidal, or organic), different charge carriers will exist. The simplest case is that of ionic salts. Here, the anions and cations carry the charge in the presence of the electric field. These typically are the most conductive types of inks, both for salt solutions and ionic liquids (ILs).^[^ ^84^ ^]^ Metal nanoparticle‐based inks are also very conductive, due to the free flow of charge that can be achieved between individual particles. However, not as conductive as bulk metal, it is possible to obtain excellent conductivity levels with these inks. Conductive polymer inks behave slightly differently. The mechanism by which charge moves depends on the structure of the polymer chain or the additives introduced to the polymer to induce conductivity. In a book chapter, Dai describes four types of conducting polymers.^[^ ^85^ ^]^ These four types are conjugated conducting polymers, charge transfer polymers, ionically conducting polymers, and conductively filled polymers. We describe the first and fourth types briefly, as they are most commonly found in ink systems.

Conjugated conducting polymer inks are the most common (e.g., polythiophene, polyaniline, polyacetylene), and have been the subject of much investigation and optimization. They are intrinsically conductive due to the overlap of π‐orbitals, which provides a pathway for charge carriers to move. To improve their conductivity, however, they are usually doped (different methods exist), as they do not have intrinsic charge carriers. Doping them also allows for increased solubility, especially in polar solvents such as dimethyl sulfoxide or water, thus allowing them to be used as inks. An example of this is reported by Cao et al. where they systematically dissolve doped polyaniline in various organic solvents and study the conductivity of the films formed from each solution.^[^ ^83^ ^]^

Conductively filled polymers comprise insulating polymer scaffolds which have been filled either with conjugated conducting polymers or other conductive solids. Also, the solvents in which they are dissolved play a great role in ensuring their conductivity. For example, the majority of polymers used in electrospraying are insulating (e.g., polystyrene, poly(methyl methacrylate), etc.). By adding metal nanoparticles, codissolving with conducting polymers, or using polar solvents, jetting can be achieved with these materials too. With this kind of modification, a great range of applications can be achieved.^[^ ^86^ ^]^

### 2.6 Relaxation Times

Lee et al. describe a set of dimensionless values which they use to optimize the experimental parameters of EHD printing regimes.^[^ ^28^ ^]^ Looking specifically at conductivity, they define a parameter *τ*_q_/*τ*_H_, which considers the charge relaxation time (*τ*_q_) and the hydrodynamic relaxation time (*τ*_H_). Hydrodynamic relaxation time refers to the time it takes for the liquid to respond (equilibrate) under new conditions. In this case, how long it takes to respond to the presence of an electric field, and is given by Equation (9).

(9) $$\tau_{H}=\frac{\eta r}{\gamma }$$

where *η* and *γ* are the viscosity and surface tension of the liquid, respectively, and *r* is the outer radius of the capillary used.^[^ ^77^ ^]^

Charge relaxation time is more related to the conductivity and not the viscosity, and is given by Equation (10)

(10) $$\tau_{q}=\frac{\varepsilon_{o}\varepsilon_{r}}{K}$$

where *K* is the electrical conductivity (S/m), *ε*_r_ is relative electrical permittivity, *ε*_o_ is vacuum permittivity (8.8542 × 10^12^ F m^−1^).^[^ ^77^ ^]^ It defines the characteristic time of charge transport determined by the electrical properties of the material.^[^ ^28^ ^]^

**Figure 5:**
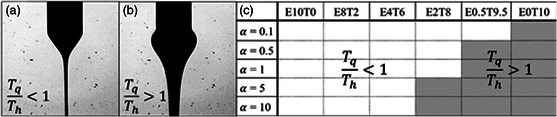
Influence of relaxation times. Representative shape of a) cone jet (τ_q_/τ_h_ < 1) and b) ball cone (τ_q_/τ_H_ > 1). c) Jetting window with respect to α for each fluid; the white region is for a classical EHD system, and the gray region is for a forced jet system (E is ethanol and T is terpineol). Reproduced with permission.^[^ ^28^ ^]^ Copyright 2013, American Chemical Society.

If the ratio is less than 1, meaning that the fluid has sufficient charge carriers and high enough electrical mobility, and the fluid is flowing slowly as in a quasiequilibrium state, then jetting can be expected (**Figure** **5**a). *τ*_q_/*τ*_H_ > 1 is characteristic of dielectric liquids (*K* ≈ 1e^−10^ S m^−1^) and does not exhibit classical jetting, but rather ball‐cone jet (Figure 5b).^[^ ^28^ ^]^ This is a mode where the flow of fluid is faster than the charge transport to the normal of the ink–air interface. This results in the electrical stress taking time to become apparent.^[^ ^78^ ^]^ This is true for highly conductive, nonviscous solutions such as poly(3,4‐ethylenedioxythiophene)‐poly(styrenesulfonate) (PEDOT:PSS).^[^ ^87^ ^]^

In any experimental arrangement, there exists a system threshold. Suitable engineering and practical design can help relax these, but it really comes down to a fundamental operational understanding of the process to maximize the output and performance. This is highlighted in work by Hayati et al. where they demonstrate that there is an optimum conductivity where a jet is stable for a fixed operating conditions.^[^ ^88^ ^]^ This stability can be achieved for other values of conductivity, if the system is adjusted accordingly.

Summarizing the aforementioned, conductivity is dependent on the charge carrier density of the ink. It can be altered by adding ionic material such as acid or salt solutions, without changing the functionality of the ink significantly. Certain solvents can also enhance the conductivity, especially in polymer inks. If the ratio of *τ*_q_/*τ*_H_ is favorable, good printing can be expected. If unfavorable, this will lead to difficulty in getting the process to work. A property which links up with the mobility of the conductive species is discussed in the following section.

### 2.7 Viscosity

Viscosity (*η*) is a measure of how resistive a liquid is to flow, due to intermolecular forces within the liquid. By definition, it is the ratio of the shear stress (*τ*) to the shear strain rate ($\dot{\gamma }$). If the viscosity is independent of the strain rate, the liquid is linear, or Newtonian. If is it dependent, it is non‐Newtonian, and requires more complex analysis. In printing systems, the viscosity of inks is commonly controlled by adjusting printhead temperature. This is because of the dependency of viscosity on temperature.

The Ohnesorge number has been used to relate the viscosity to the surface tension in the following relation (Equation (11)).

(11) $$O_{h}=\frac{\eta }{\sqrt{\rho \gamma L}}$$

where *η* is the ink's viscosity, *ρ* is the density, *γ* is the surface tension, and *L* is the droplet length (usually the drop diameter). A more useful parameter however is *Z*, the reciprocal Ohnesorge number. It has been found that if *Z* lies between 1 and 10, jetting can occur. If *Z* is below 1, viscous forces dominate, and no droplets can form. When *Z* is greater than 10, the droplets could break into satellite droplets, thus reducing the printing quality considerably.^[^ ^89^ ^]^ This parameter, along with the Weber number (We) and Reynolds number (Re), have been extensively used in determining ink printability and other properties for inkjet processes, but less so for EHD. Few studies which focus on EHD report these values, such as the work by Zhang et al.^[^ ^78^ ^]^ These numbers provide a reliable framework from which experiments can be designed.

Viscous inks include polymers, ILs, gels, oils, and organic solvents like glycerol. Higher viscosity inks have been demonstrated to form more stable jets during EHD printing, and hence more reliable resolution, so long as *Z* < 10, and the conductivity is sufficient.^[^ ^25^, ^90^ ^]^ Polymer material, such as polyvinylpyrrolidone (PVP) has been used to increase the viscosity of inks whose viscosity was too low.^[^ ^91^ ^]^ Increased ink viscosity also contributes to a reduction in the coffee ring effect during feature drying, especially where colloidal suspensions are printed. This was demonstrated by Tu and Lee where they added 2 wt% dimethyl sulfoxide to a solution of PEDOT:PSS, and found that the resulting thin films formed were smoother and more uniform.^[^ ^92^ ^]^ With regards to response to electrostatic charge or potential bias, the hydrodynamic relaxation time is directly proportional to the viscosity (Equation (9)).^[^ ^77^ ^]^ In other words, higher viscosity translates to higher hydrodynamic relaxation time.

According to Equation (9) and (10), droplet formation time increases with higher viscosity and decreases with higher surface tension or higher conductivity (i.e., either the droplet formation is driven by charge build up or by fluid flow). Therefore, for successful EHD printing, it is important to minimize the viscosity to ensure a short *τ*_q_, while keeping the surface tension and conductivity relatively high for a fixed stand‐off height and nozzle width, but keeping in mind the formation of satellite droplets because of Rayleigh breakup.^[^ ^42^ ^]^ Phung et al. make recommendations for what the viscosity values should be for inks, depending on the mode of EHD being carried out.^[^ ^93^ ^]^ Barrero et al. reported that experiments have shown that a cone‐jet mode of EHD is only possible if $\tau_{q}$ < $\tau_{H}$.^[^ ^29^ ^]^ This is corroborated by Choi et al. and Edirisinghe et al. in their work.^[^ ^94^, ^95^ ^]^ Further, they reported that for pulsed mode jetting, the pulse used should be greater than the hydrodynamic relaxation time and the Rayleigh time (i.e., the minimum time required to form a stable jet) for stable jets to be produced.

### 2.8 Ink Flow Rate

A parameter which is usually left unreported in most EHD work, has been the flow rate. When analyzing the force diagram of the EHD jetting phenomenon (Figure 1a), it is critical to remember that a back pressure is required to continuously supply fluid to the capillary tip for an equilibrium to be maintained. This pressure is maintained either by a static pressure, as in the work by Poellmann et al.,^[^ ^96^ ^]^ or a continuous flow of ink from a reservoir. It has been reported that the hydrodynamic relaxation time is also related to flow rate by the following Equation (12)^[^ ^95^ ^]^

(12) $$\tau_{H}=\frac{LD^{2}}{Q}$$

where *D* is the jet diameter, *Q* is the flow rate, and *L* is the axial length of the jet.

**Figure 6:**
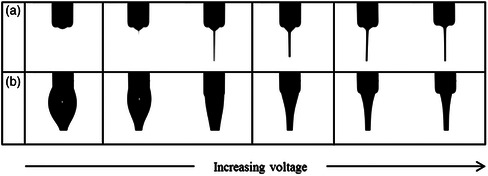
Evolution of cone jet of polyethylene oxide (PEO) (M_w_ = 1.0 × 10^6^ g mol^−1^, 0.38 wt%) in water/glycerol mixture: a) thin jet from the insufficient flow rate and b) thick jet from the excessive flow rate. Reproduced with permission.^[^ ^46^ ^]^ Copyright 2016, Elsevier.

Jet stability has been reported to increase (to a maximum value) as the flow rate increases for a given capillary diameter, conductivity, and applied voltage, but this results in much larger droplet sizes forming, thus reducing the resolution of printing that can be obtained.^[^ ^43^, ^97^ ^]^ If the flow rate is increased to be much more than the jetting frequency, satellite droplets, and large drops are likely (as *τ*_q_/*τ*_H_ > 1). If the opposite is true, the printed features can either be discontinuous or vary significantly in width. Work by Scheideler and Chen on the scaling of Taylor cone jets introduces new understanding for high viscosity fluids. They propose that the minimum flow rate, *Q*, required to sustain a stable Taylor cone scales to the viscous diffusion time as given by *Q*_m_ ~ *γD* ^2^/*η,* where *D* is the nozzle outer diameter.^[^ ^81^ ^]^ A similar relationship is observed if Equation (9) and (10) are combined and expressed in terms of *Q*. **Figure** **6** shows the problem of poor jetting as a result of a) insufficient flow rate and b) excessive flow rate, as reported by Yu et al.^[^ ^46^ ^]^

Similarly, for low viscosity liquids, with high conductivity, Barrero and Loscertales reported a minimum flow rate *Q*_min_ which scales as $Q_{\text{min}}∼\frac{\varepsilon_{r}\gamma }{\rho K}$, where *ε*_r_ is the permittivity of the ink, *γ* the surface tension, *K* is the conductivity, and *ρ* is the density.^[^ ^30^ ^]^ Knowing which regime each ink is in can assist EHD users in understanding better the observed behavior and how to achieve their desired outcome.

### 2.9 Ink Development for EHD

ILs fulfil the criterion for a good ink. A significant drawback though of ILs is that they do not evaporate readily because of a negligible vapor pressure^[^ ^98^ ^]^ and high viscosity.^[^ ^99^ ^]^ This would severely limit the application of these as ink solvents in conventional applications, but potentially open new avenues of research as has been shown in their application in biomaterial delivery.^[^ ^99^, ^100^ ^]^ Wang et al. demonstrated the first DOD use of printed perovskite IL to create a photodetector.^[^ ^101^ ^]^

Small organic molecules which fluorescence can be printed as ink when dissolved in the right solvent. They can also be mixed with a variety of other materials and can be utilized as an imaging tool in the printing process. The quest to achieve ever higher resolutions will also require that better metrology of printed features be performed. Scanning electron, confocal fluorescence, and atomic force microscopy are tools which could be utilized extensively to measure accurately the printing sizes achieved. Work incorporating fluorescent molecules as markers has not been fully developed. Sutanto et al.^[^ ^102^ ^]^ demonstrated its capability. However, they have been investigated for use as organic light‐emitting diodes.

Liquid crystals (LCs) are a type of mesophase material in that they possess the anisotropic properties of solids, and the fluid character of isotropic liquids. This makes them sensitive to external field perturbations (electric, mechanical, magnetic, and thermal). They have been shown to be good hosts for nanomaterials such as nanoparticles, quantum dots, bionanostructures, etc. With this in mind, high‐resolution printing of these hybrid structures could lead to very interesting applications in optoelectronics.^[^ ^103^ ^]^ Currently, LCs have been aligned for applications using several contact and noncontact methods including the rubbed polymer technique, nanoimprint lithography, photoalignment, and ion beam alignment.^[^ ^104^ ^]^

LCs, due to their unique properties, are likely to be able to act as good inks in the field of EHD, but no literature has successfully demonstrated this explicitly. Byun et al. used EHD (multimode printing) to deposit polyimide onto a substrate for LC alignment, but did not print the LC itself.^[^ ^105^ ^]^ This presents a great opportunity to build up this body of knowledge from a high‐resolution printing perspective.

## 3 System Parameters

The specific configuration of EHD printers is highly variable. Commercially bought systems can offer some kind of uniformity in performance, but most researchers develop their own in‐house systems. This is an excellent occurrence as it means that the technique is more accessible to a wider audience. However, every researcher will have different objectives, thus changing how their printers are put together. Factors such as the nozzle size, stage speed, applied voltage are all variable. This section describes the relationship between these variables and **Table** **4** shows key one. Several groups have conducted studies to quantify these relationships, and their results are corroborated by our own unpublished observations.^[^ ^106^, ^107^ ^]^

**Table 4:** Illustration of the relationship between various system variables at play during EHD printing

Constant	Independent	Dependent	Relationship
Flow rate	Stand‐off height	Critical voltage	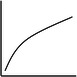
Stand‐off height	Flow rate	Critical voltage	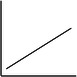
Flow rate Stand‐off height	Applied voltage	Line width	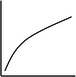
Applied voltage Stand‐off height	Flow rate	Line width	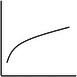
Applied voltage Stand‐off height	Nozzle diameter	Line width	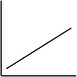

### 3.1 Nozzle Diameter, Geometry, and Arrangement

**Figure 7:**
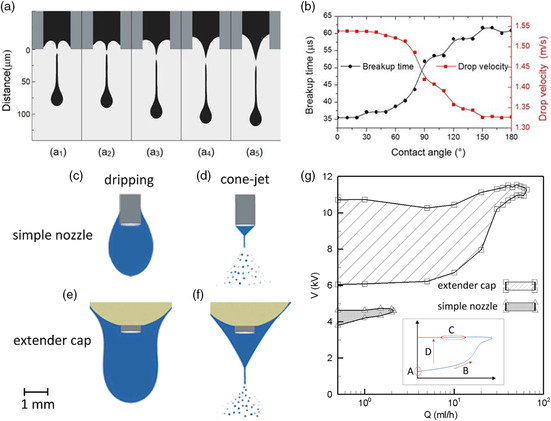
The nozzle wettability influences. (a) The ink droplet image at the moment of breakup. The contact angles of the nozzle inner wall and the breakup times are (a_1_) θ = 30°, 37.14 μs; (a_2_) θ = 60°, 38.78 μs; (a_3_) θ = 90°, 51.89 μs; (a_4_) θ = 120°, 58.45 μs; (a_5_) θ = 150°, 61.73 μs. (b) The breakup time and droplet velocity changing with the wettability of the nozzle inner wall. a,b) Reproduced under the terms of the CC‐BY 4.0 license.^[^ ^44^ ^]^ Copyright 2017, The Authors, published by Springer Nature. The liquid rises the wettable nozzle outer wall in the dripping mode (c). After installation of the extender cap, the recess was small enough for rising droplet to reach the extender cap and adhere to its bottom surface (e). When the electric field is intensified the cone is formed with its base connected to the extender cap (f), instead of the nozzle tip itself (d). (g) The shaded regions show stability islands corresponding to the two nozzles. The inset shows a typical stability island with the corresponding zones. c–g) Reproduced under the terms of the CC‐BY 4.0 license.^[^ ^43^ ^]^ Copyright 2016, The Authors, published by Springer Nature.

The influence of the nozzle diameter on a variety of parameters or outcomes can be significant.^[^ ^108^ ^]^ The final resolution obtained with EHD printing can be determined in the most part by this. Park et al. are the first to state that the nozzle diameter is a massive limitation.^[^ ^2^ ^]^ Schneider's pioneering work on the formation of out of plane nanopillars by nanodroplet autofocusing could not have been possible without the use of very small capillary nozzles (≈ 500 nm).^[^ ^20^ ^]^ In addition to size, it has been demonstrated that the coating of the outside of conductive nozzle with a hydrophobic self‐assembled monolayer reduces the meniscus size, thus promoting finer jets.^[^ ^96^ ^]^ Work done by Stachewicz et al. has demonstrated the significant difference in jetting when a capillary with no monolayer is used.^[^ ^109^ ^]^ Using pristine nozzles (i.e., no surface energy modification agent), the droplet formed on the nozzle can be effectively bigger than the nozzle as it climbs up the outer surface by capillary action, akin to the ball‐cone jet. This, in turn, makes the jet diameter larger than what it would have been if the meniscus had been limited to the true nozzle diameter. Looking at an example found in an inkjet printing study, He et al. studied the influence of wettability within the capillary itself (**Figure** **7**a,b).^[^ ^44^ ^]^ By increasing the hydrophobicity of the inner wall, they observe that droplet breakup occurs at a later point than that observed for a hydrophilic wall. This leads to a longer filament being formed, thus increasing the probability of stray satellite droplets forming. A hydrophilic inner wall, however, exhibits stronger adhesion to the ink, thus promoting shorter filament lengths, and quicker droplet breakup—giving better printing quality overall for the studied ink. Morad et al. discussed using extender caps on capillaries to enhance the Taylor cone stability (Figure 7c,d).^[^ ^43^ ^]^ They attached a hemispherical cap to the end of a stainless steel capillary, which can be wet by the ink being investigated. With this modification, they demonstrate a larger island of Taylor cone stability, thus allowing for a wider range of applied potentials as a function of the flow rate used (Figure 7e–g).

**Figure 8:**
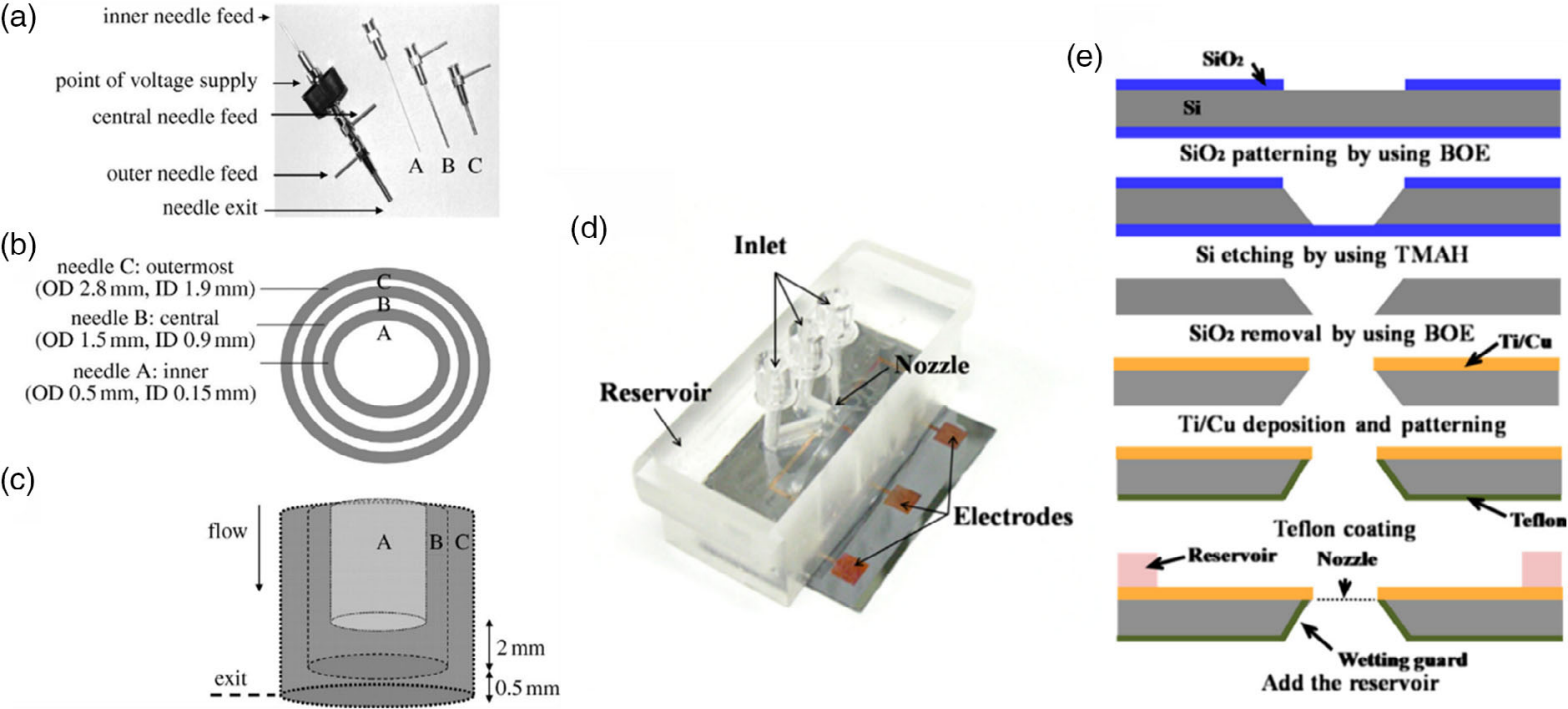
Details of three‐needle coaxial device: (a) needle assembly, (b) needle dimensions and (c) relative placement of needles in device. ID and OD represent internal and outer diameters, respectively. a–c) Reproduced with permission.^[^ ^116^ ^]^ Copyright 2008, The Royal Society. (d) Fabricated multi‐nozzle and (e) fabrication flow chart of the multi‐nozzle. d,e) Reproduced with permission.^[^ ^120^ ^]^ Copyright 2008, AIP Publishing.

Coaxial needle geometries have also been explored, especially for biomedical applications, the formation of core–shell particles^[^ ^110^, ^111^, ^112^ ^]^ or fibers^[^ ^113^ ^]^ and hollow nanofibers.^[^ ^114^ ^]^ The inner nozzle is used to extrude a different material to that which flows through the outer nozzle. A very high‐resolution 40 nm lead zirconate titanate (PZT) device was drawn using this configuration by Wang et al.^[^ ^115^ ^]^ They used silicone oil as the outer material, and PZT as the inner material. The enhancement in resolution is achieved by the compression offered by the outer material in the jet. It is imperative that the two materials be insoluble, and of different viscosities to achieve the high resolution desired. Ahmad et al. reported a trineedle coaxial arrangement which they used to form nanocapsules, layered bubbles, and porous encapsulated threads, with the intention of eventually developing capsules for multistage drug delivery (**Figure** **8**a‐c).^[^ ^116^ ^]^ Several reviews exist which summarize specifically this kind of work.^[^ ^117^, ^118^ ^]^ Multinozzle geometries have also been developed. Traditionally, nozzles used in EHD are needle shaped. This is due to their availability and ease of use. Sutanto et al. produced an industrial system, which consisted of multiunit print heads.^[^ ^102^ ^]^ They also demonstrated a three nozzle system which they use to print micro‐optical devices.^[^ ^119^ ^]^ A multinozzle arrangement helps to improve the relatively low throughput seen in EHD printing, and makes the technique easier to use in large‐scale production. There can be, however, issues of interference between each nozzle in the electric field, leading to poorer printing quality and misalignment of structures. To overcome this challenge, Lee et al. designed and fabricated a multinozzle planar device from a silicon wafer. The spacing of the wafers, and their design, contributed successfully to producing individually controlled stable jets which showed no signs of distortion in the electric field. By designing their system as a microfluidic device, they were also able to control effectively the flow rate such that the jetting voltage could be kept constant (Figure 8d,e). A wetting control was also used (Teflon) to prevent excessively large pendant drops from forming.^[^ ^120^ ^]^

Nozzles can also be completely omitted. Nozzle‐less printing can be achieved using alternate actuation methods. Here, Coppola et al. has done significant work in demonstrating the use of pyro‐EHD.^[^ ^121^, ^122^, ^123^ ^]^ Pyro‐EHD makes use of a ferroelectric crystal which, when heated, generates an electric field. This field then induces jetting of the ink. Microscale resolution printing has been achieved using pyro‐EHD, creating devices such as optical microlenses.^[^ ^124^ ^]^

Concluding, the enhancement of the resolution obtained in EHD printing can be promoted by customizing the nozzle size or geometry and using common surface modification methods. Depending on how fast the process is intended to be, introducing multinozzle heads can increase the throughput significantly.

### 3.2 Applied Potential, Tip‐Substrate Separation, and Operating Conditions Influence on Resolution

The applied potential and stand‐off height control the magnitude of the electric field generated, directly and indirectly, respectively. Computational models have been developed to understand the relationship between these two parameters.^[^ ^125^ ^]^ The stand‐off height needs to be large enough to allow for the formation of a jet, whilst being small enough to not allow droplet pinching over time as the jet starts to disintegrate if the stable cone jet is the mode desired, and for a constant potential. It has been demonstrated too that a stand‐off height that is too large (for a low applied potential) can facilitate increased wetting as a result of splashing.^[^ ^19^ ^]^

**Figure 9:**
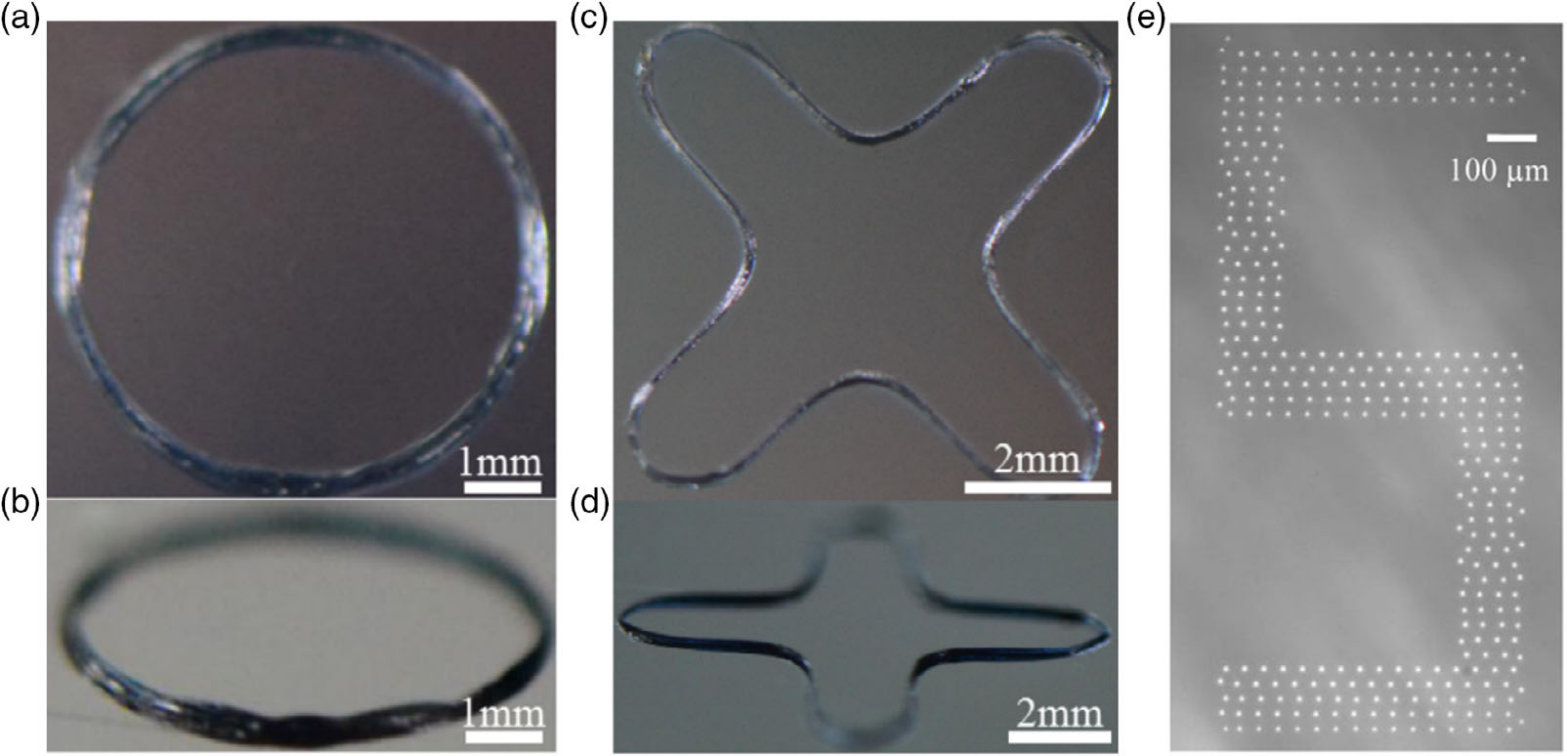
EHD printing of multilayer curved PEDOT:PSS‐PEO features. a,b) Optical photos of circular features with a layer number of 200. c,d) Optical photos of 300‐layer curved features. a–d) Reproduced with permission.^[^ ^127^ ^]^ Copyright 2018, American Chemical Society. e) Drop‐on‐demand printed “S” on glass slides with printing speed and droplet dimension controlled by the parameter of the AC‐pulse voltage. Reproduced with permission.^[^ ^130^ ^]^ Copyright 2014, IOP Publishing.

As already mentioned, there exist several modes of stable EHD printing. Continuous jet printing, as the name suggests, involves a continuous flow of material from the nozzle onto the substrate.^[^ ^126^ ^]^ The applied potential is kept constant such that the ink continues to eject uninterrupted until the desired pattern is achieved (**Figure** **9**a‐d).^[^ ^127^ ^]^ This mode is most commonly achieved with a DC voltage. The balance between flow rate and applied bias must be kept in order for regular printing to be achieved. A discrepancy in this balance can lead to broken patterns, bulging or a splurge of material which no longer resembles printing. The speed of the translational stage on which the substrate sits can aid in achieving high‐quality continuous printing. This has been studied in‐depth by Phung et al.^[^ ^91^, ^93^, ^128^ ^]^ They demonstrated that if the speed is too slow, excessive deposition occurs. However, if the speed is too great, then discontinuities occur due to the deposition being too slow. In our own work, we demonstrated this where we printed (3‐aminopropyl)triethoxysilane onto a silicon wafer. Where the speed of the stage was accelerating, a higher resolution was obtained (i.e., narrower line width). Where the speed was slow, a wider line width was obtained.^[^ ^129^ ^]^

DOD printing is a mode where drops are deposited in precise positions by the application of a jetting pulse (Figure 9e).^[^ ^130^ ^]^ This mode is generally achieved using superimposing AC bias over a constant DC bias, but can also be achieved using DC voltage alone in an unsteady mode. If the interdroplet distance is small, coalescence of the droplets is observed and a continuous print pattern can also be achieved.^[^ ^56^ ^]^ Generally however, DOD EHD is used to generate patterns where discrete points are advantageous. Park et al. demonstrated DNA‐directed nanoparticle assembly and DNA‐aptamer biosensing based on DOD‐printed patterns.^[^ ^131^ ^]^ Zhang et al. presented 3D‐printed nanostructures with aspect ratio over 35 printed using DOD EHD.^[^ ^66^ ^]^ The pulse frequency and pulse duration control the resolution in this mode of printing. Laurila et al. presented an analysis of the relationship between the pulse frequency, duration, duty cycle, and applied potential.^[^ ^51^ ^]^ In their investigation, they determine that the droplet size has a negative relationship with the pulse frequency (i.e., low‐frequency results in large droplet size). This is explained by the relationship between charge build‐up and droplet pinch off. If the meniscus has a longer time to build up charge, it means that the droplet which will pinch off will be of a greater volume. It follows, then, that for a large pulse width, a large droplet size is observed. Combining a high pulse frequency with a low stage speed results in coalescence of the deposited features. Thus, if small discrete drops are required, with a high resolution, then the pulse frequency must be high, the pulse width low and the translation speed high too, as was achieved by Zhao et al. where they printed graphene as microelectrodes.^[^ ^132^ ^]^

As previously mentioned, the applied potential needs to supply enough electric stress to overcome the surface tension while not providing excessive charge, which would lead to multiple jets being formed.^[^ ^43^ ^]^ The variety of jetting modes which can arise because of different applied potential conditions has been extensively documented.^[^ ^6^ ^]^ The majority of EHD studies utilize DC voltage to induce printing. If performed onto insulating surfaces, this can lead to poor resolution as the ink droplets would be highly charged, and therefore, repel each other. Wei et al. introduce an AC‐modulated EHD process which can achieve good printing, by overcoming the above limitation.^[^ ^130^, ^133^ ^]^ Using high dielectric constant inks also increases the risk of charge build‐up and breakdown. To prevent this, more sensitive inks are printed in vacuum or inert gases such as nitrogen or argon. This kind of printing requires more resources than what is conventional for EHD, and so comes with a slightly higher cost.

Sammons et al. present one of the first investigations into how humidity and environmental temperature affect the EHD process.^[^ ^26^ ^]^ By assessing the quality of the printed adhesives they studied in different conditions, they concluded that humidity had a minor effect on the overall printing behavior, for the material used in their study. However, temperature had a more pronounced influence, resulting in printed droplet diameter increasing as temperature increased. They also noted that at higher ambient temperature, the initial jetting voltage decreased, as did the jetting frequency. This correlates with the discussion earlier in this work regarding surface tension and temperature. Ongoing investigations into all these factors will inform better‐quality printing. Their observations regarding humidity, however, are not true in general. Increasing the humidity greatly reduces the chance of solvent evaporation, thus giving any printed structure time to spread. This was demonstrated by Lee et al. where they dispensed microarrays of DNA droplets and monitored the drying.^[^ ^134^ ^]^ They found positive correlation between the increase in humidity and an increased spot size. Further, an increased relative humidity results in longer jet formation times. As the charge builds up on the meniscus, there is a greater chance of it dissipating into the surrounding air, thus delaying the deformation of the meniscus by electrostatic forces. It is therefore recommended to keep relative humidity reasonably low, or to use inert gas environments to overcome these challenges.

## 4 Substrate Considerations

Many different substrates have been used for EHD printing experiments. They include glass, PDMS, silicon dioxide wafers, flexible polymers such as polyethylene terephthalate (PET), paper, metal‐coated surfaces, among others. The major determining factor for the substrate choice is the end‐use of the printed structures. Factors such as processability for electronic devices, or biocompatibility for biological applications are key. Irrespective of the application, the surface plays a big role on the success of EHD printing, thus the following considerations must be considered.

### 4.1 Chemical Control

**Figure 10:**
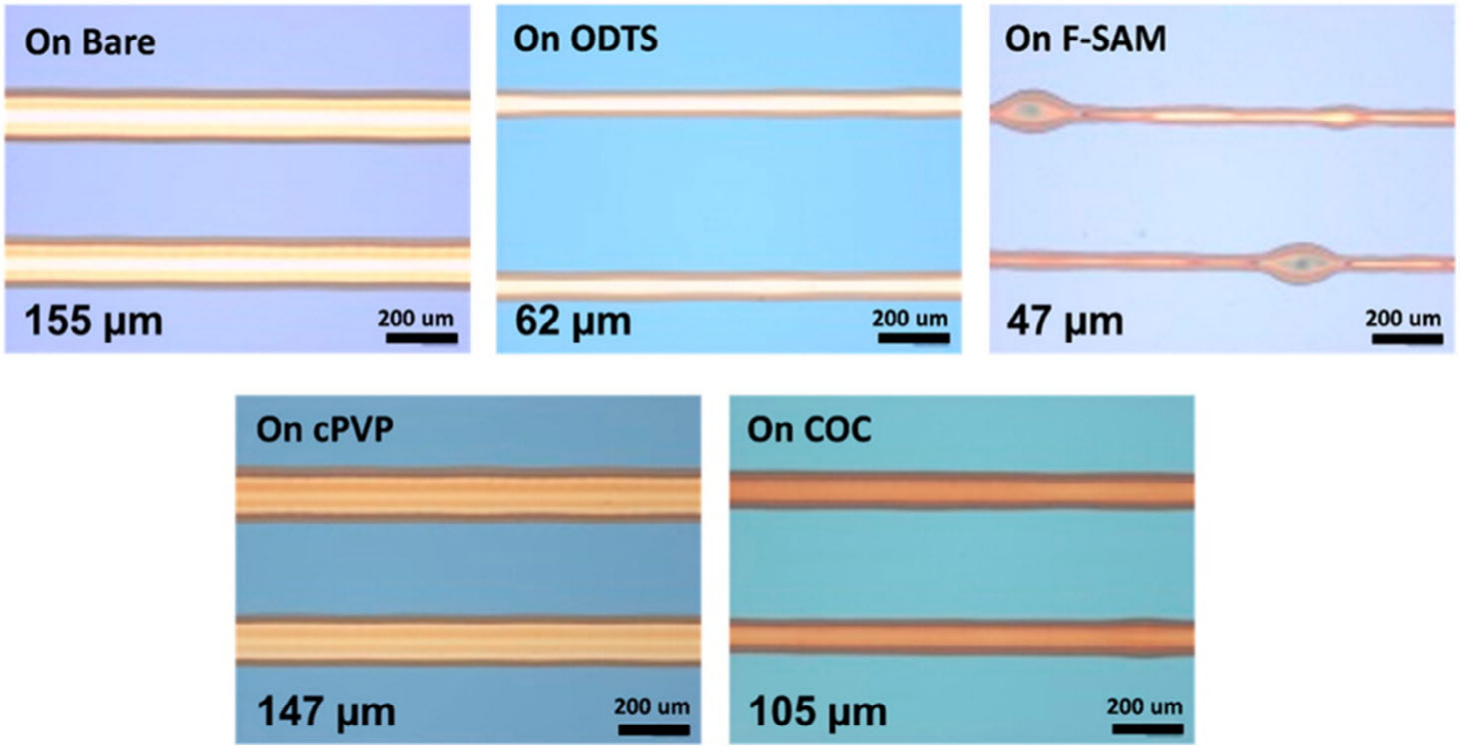
Optical images of the P3HT lines on differently modified surfaces. Reproduced with permission.^[^ ^136^ ^]^ Copyright 2014, American Chemical Society.

Control of printing resolution does not only depend on the actuation method, but also on the chemistry of the surface being printed on. The chemistry of the surface determines surface energy. High energy surfaces (*θ*_C_ ≤ 5°) are highly wettable. This means that any printed material will spread extensively to minimize the potential energy. A well‐known method of controlling the surface energy of surfaces is using self‐assembly monolayers, which impart hydrophobicity or hydrophilicity depending on their terminal chemistry. A detailed look through published reports reveals that the most commonly used are thiol‐based, silane‐based, or halogenated^[^ ^135^ ^]^ moieties. Jeong et al. investigated two such monolayers, as well as polymer thin‐film coatings to assess the effect on the printing morphology and resolution.^[^ ^136^ ^]^ They utilized Si wafers with a 300 nm SiO_2_ layer which had been cleaned in hot piranha solution. This hydroxylated the surface and prepared it for further modification. The monolayers assessed were 1H,1H,2H,2H‐perfluorooctyltriethoxysilane (F‐SAM) and octadecyltrichlorosilane (ODTS) in toluene. The polymers (poly(4‐vinylphenol) and ethylene‐norborene cyclic olefin copolymer) are commonly used as coatings for electronic devices due to their thermal stability. They reported the smallest print widths were obtained with F‐SAM (due to the polarity of the fluoro groups), but they observed periodic bulging in the printed lines (**Figure** **10**). They attributed this not to surface chemistry, but rather to an over delivery of ink to the tip (excessive flow rate). This was overcome by speeding the substrate stage up during printing to allow for less ink accumulation on a particular spot. In essence, what they found was that as the surface energy decreased, the width of the printed lines also decreased compared with lines printed on a bare substrate. However, the authors did not explicitly measure the thickness of the printed lines, it could be hypothesized that the narrower lines were thicker than the wider lines—if the jetting frequency were the same. Interestingly, when they carried out electric characterization of the printed lines, they found that the lines printed on the ODTS surface carried out better every time due to a higher order of crystallinity imparted by the ODTS on the ink, poly(3‐hexylthiophene) (P3HT).

**Figure 11:**
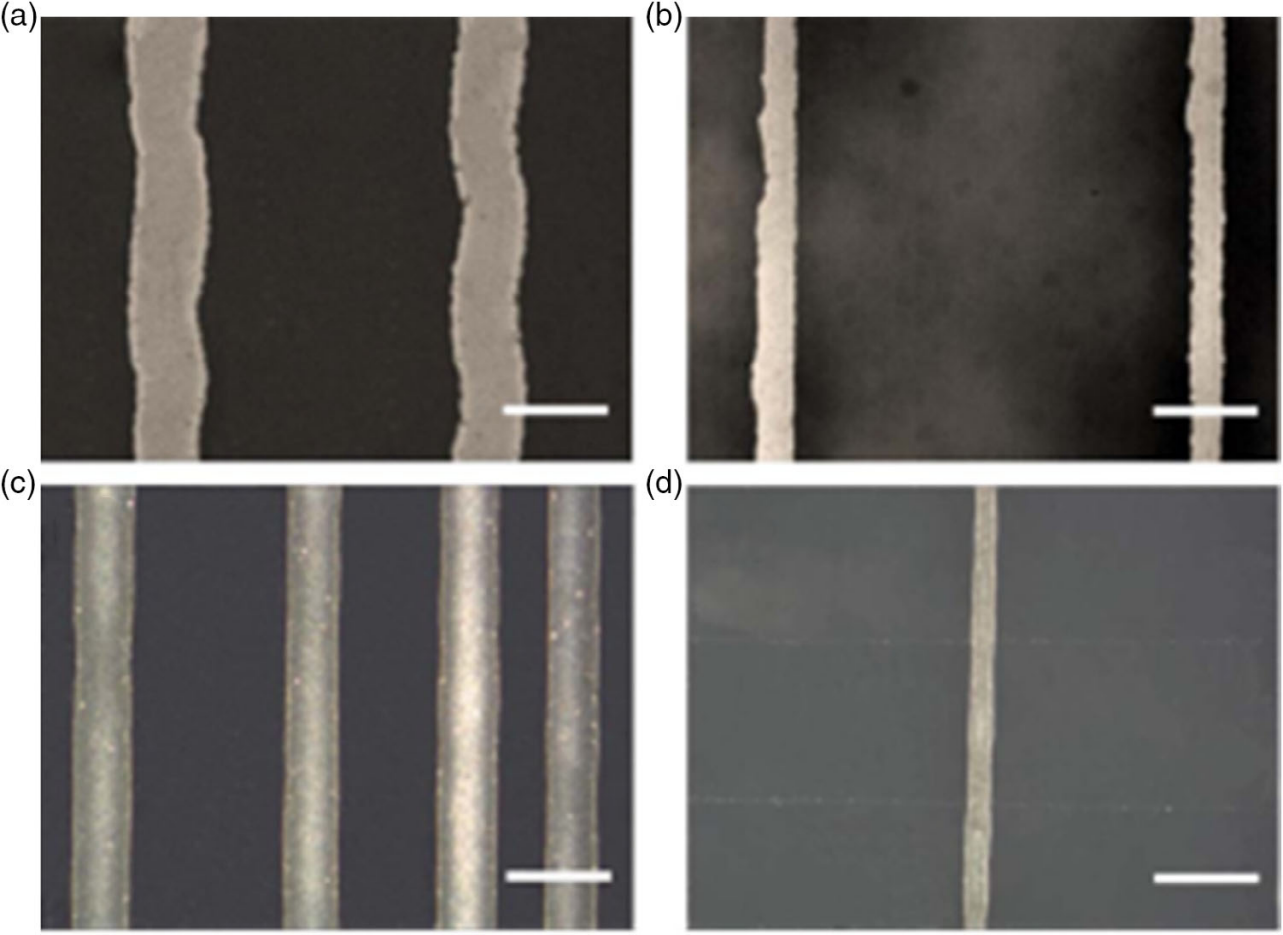
Optical images of printed Ag lines. Printed Ag nanoink on (a) PET film and (b) PEN film. Printed Ag nanopaste on (c) PET film and (d) PEN film. Scale bars are 100 μm. Quality of printing appears better on PEN than on the PET. Reproduced under the terms of the CC‐BY 4.0 license.^[^ ^137^ ^]^ Copyright 2015, The Authors, published by ECS.

In a similar study, Kwon and coworkers showed just how much the surface energy has a role to play in the wetting behavior of solvents used (**Figure** **11**).^[^ ^137^ ^]^ They compare a low viscosity silver ink with a silver paste on several surfaces including glass, PVP‐coated glass, PET, and polyethylene naphthalate (PEN). They utilized an AC voltage (which aided in overcoming the bulging issue observed by Jeong et al.^[^ ^136^ ^]^) to achieve printing from relatively large nozzles (25 μm for low viscosity; 50 and 100 μm for paste). By printing on a plasma‐cleaned bare glass surface (very high energy), poor resolution and adhesion were obtained for both inks. This is also due to the unwanted hydrophilicity imparted by the plasma cleaning. The PVP glass displayed better adhesion and thus better resolution. The printing on the plastic films also corroborated the idea of low surface energy leading to better resolution, as the PEN surface was better than that of the PET.

The adhesion of ink to a surface must be considered. As shown in nature, it is possible to have hydrophobic surfaces which have good adhesion and those which have poor adhesion. This is demonstrated by Bao et al. in their development of superhydrophobic yet highly adhesive surfaces.^[^ ^138^ ^]^ A commonly used test for adhesion is the Scotch tape test, where a piece of Scotch tape is placed over a feature and pulled off. If the feature stays on the substrate, it is well adhered.^[^ ^139^ ^]^

Further consideration is required for colloidal inks, as they are especially prone to the coffee ring effect, if not printed and cured in appropriate conditions. This could drastically alter the properties of the final structures printed. It has been demonstrated that on hydrophilic substrates, the particles from colloidal inks dry inhomogenously and exhibit a bimodal distribution. As the solvent evaporates, the particles aggregate near the edge of the drop or printed features due to convective capillary flow pushing them to regions of lower particle concentration. If we consider a droplet on a hydrophilic surface, it will have a large contact surface area but low contact angle (<65°). This means that the droplet dries up quickly, thus promoting rapid and poor particle assembly. However, on hydrophobic substrates, the particles do not spread out as significantly, but rather form more localized structures. Here, the droplet has a low surface area, but large contact angle (>65°). The solvent evaporates slower, giving the particles more time to assemble, resulting in more dense structures forming.^[^ ^140^ ^]^

The end application, desired feature resolution and ink type used are factors which must be considered when determining what kind of chemical functionalization a surface requires.

### 4.2 Physical Control

**Figure 12:**
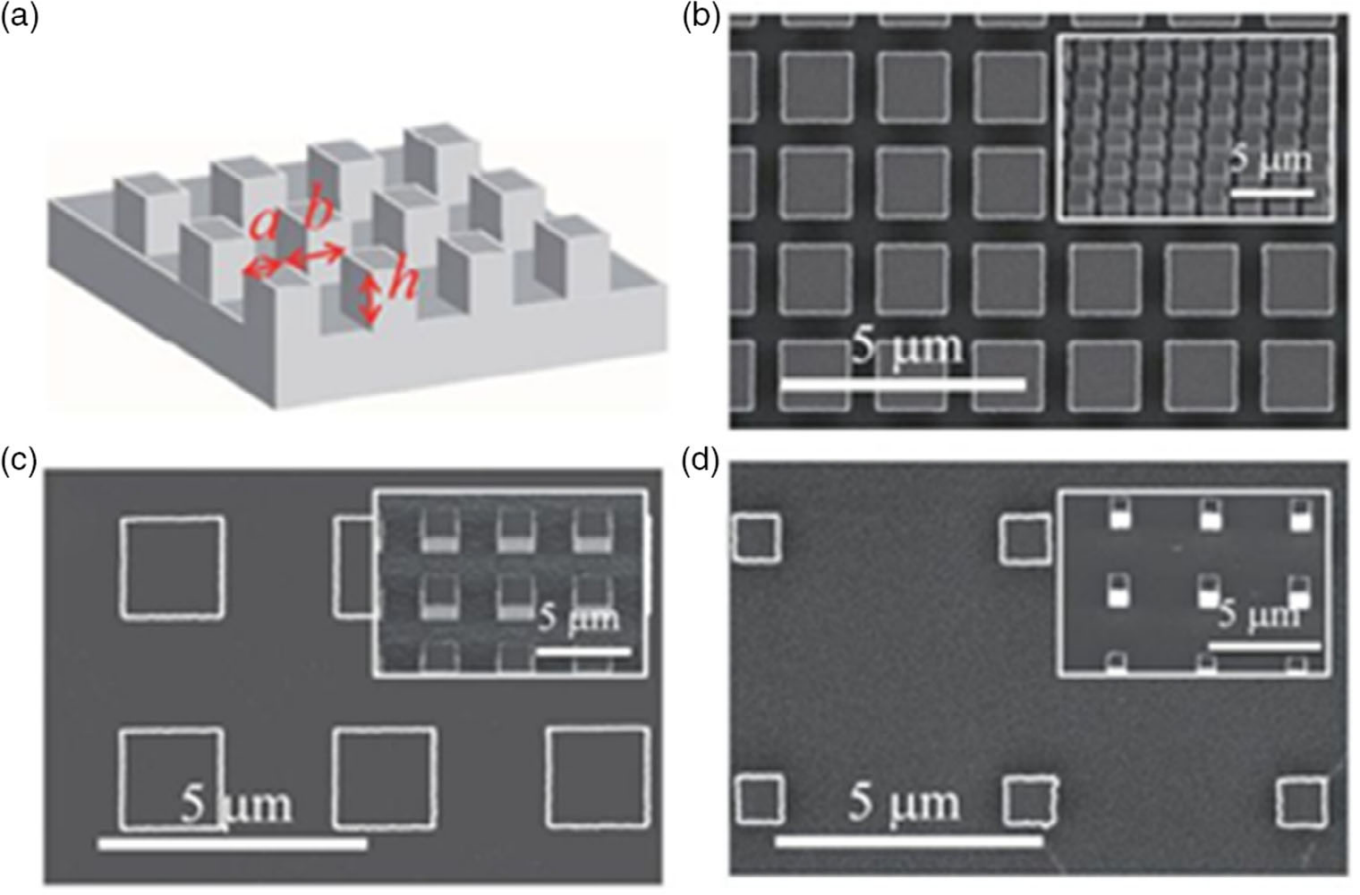
Surface features for wetting control. (a) Schematic showing terminology for pillar width a, separation b and height h. (b–d) Scanning electron microscopy images of representative micro‐structured surfaces for (b) b/a = 1/3, (c) b/a = 1, and (d) b/a = 4. Reproduced under the terms of the CC‐BY 4.0 license.^[^ ^142^ ^]^ Copyright 2015, The Authors, published by Springer Nature.

Wettability is also a function of the microscale roughness of a surface.^[^ ^141^ ^]^ For instances, where more long‐term hydrophobicity is desired, the use of physical features can be beneficial. These features can either be lithographically patterned, or introduced additively. Wang et al. demonstrated how the surface structure determines the wettability that can be obtained.^[^ ^142^ ^]^ They lithographically patterned micropillars into silicon wafers, keeping height (*h*) fixed and varying the length (*a*) and width (*b*) (**Figure** **12**). They define the roughness as [(*a* + *b*)^2 ^+ 4*ah*]/(*a* + *b*)^2^. Having patterned these features (smaller than those previously reported), they then carried out wetting experiments using three different solutions with similar surface tensions and different viscosities and compared the results with wetting on flat substrates.

They found that the surface structure geometry greatly influences the dynamic wetting. They suggested then that it is possible to control the nonequilibrium wetting behavior by appropriate surface engineering. This means that by engineering a surface to which printed structures can adhere to (by solvent front pinning), a desired resolution can be achieved.

Other means of inducing surface roughness for wetting control have been demonstrated, including the use of dense nanoparticle arrays,^[^ ^143^ ^]^ laser ablation treatment,^[^ ^144^ ^]^ polymer wrinkling,^[^ ^145^ ^]^ mechanical abrasion, vapor deposition, creating biosurface, electrochemical deposition, and others.^[^ ^146^ ^]^ The substrate being used would determine which process would be most appropriate to induce the roughness desired. By achieving control of how an ink moves or spreads over a surface, the control over resolution is achieved.

### 4.3 Electrical Control

**Figure 13:**
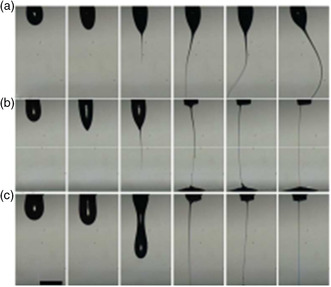
The formation process of the jet over the different substrates. The nozzle‐to‐substrate distance H is fixed at 2 mm, the flow rate is Q = 50 nL/min, and the substrate is still. a) PET. b) Silicon. c) Steel. Reproduced with permission.^[^ ^147^ ^]^ Copyright 2013, Trans Tech Publications Ltd.

The electrical properties of the surface being printed on are the final intrinsic consideration. However, they do not directly contribute to the resolution of printing obtained, they can contribute to the quality of features achieved. For instance, Bu et al. looked at the EHD process when considering an insulating, dielectric, and conductive substrate. They found that the substrate type plays no role in the jetting voltage, however, the jet character is influenced.^[^ ^147^ ^]^ **Figure** **13** shows how the evolution of the jet differs for substrates of varying electrical characteristics.

**Figure 14:**
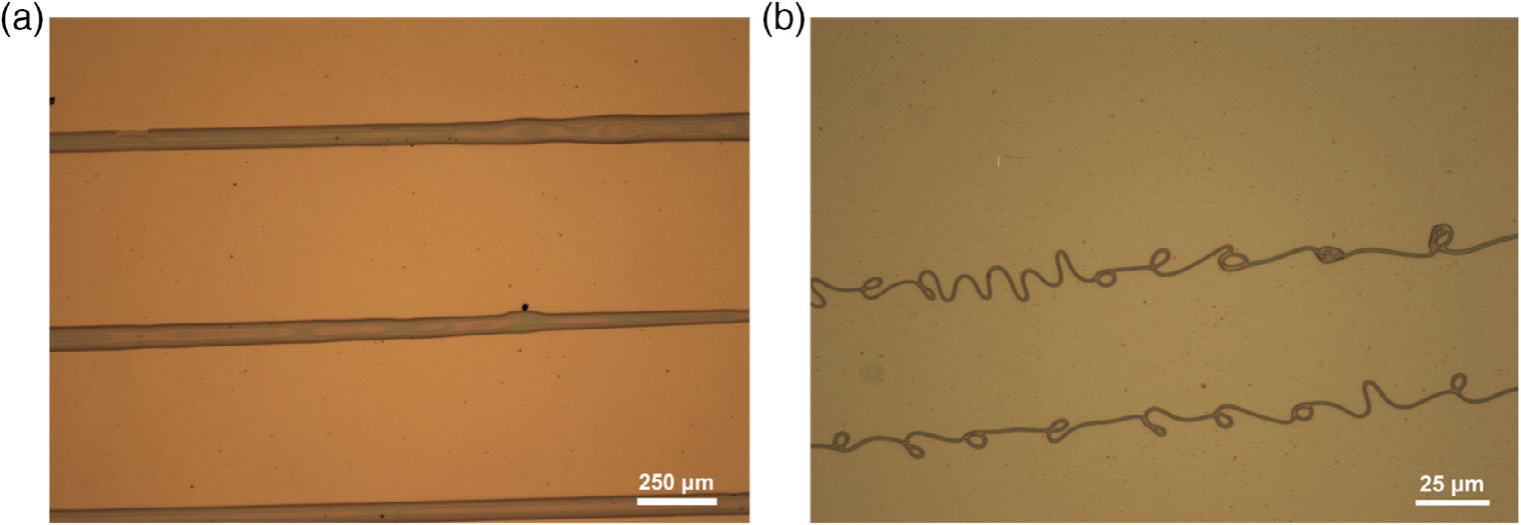
EHD‐printed PMMA. a) Regular patterns with relatively large line width due to sufficient discharge occurring before jet lands on substrate. b) Nonlinear lines due to whipping instabilities introduced by excessive applied bias.

Their work carries on to state that when the charge relaxation time of the substrate is lower than the jet lifetime, the deposited material will form uniform straight lines. However, when the substrate charge does not dissipate before the jet lifetime, whipping instabilities occur leading to disordered printing, most commonly observed for polymer near‐field electrospinning. This was observed in the author's own work when he attempted to print poly(methyl methacrylate) (PMMA) **(** **Figure** **14**). This work supports the earlier‐stated reasoning for using AC voltage on insulating substrates, to prevent charge build‐up and allowing for printing to occur.^[^ ^130^ ^]^

## 5 Outlook

As the scope of additive nanomanufacturing grows, the role of EHD printing will become ever more important, mostly due to the wide range of fields it can be used in. As this fabrication method is relatively more accessible, compared with high‐cost facilities such as clean rooms, electron beam lithography, and photolithography, among others, it will allow for more researchers to explore unique ideas, especially in developing countries.

A thorough understanding of its application will open up more creative and interesting applications including displays,^[^ ^148^ ^]^ transistors,^[^ ^149^ ^]^ lithography,^[^ ^21^, ^150^ ^]^ nano electromechanical systems (NEMS)/micro electromechanical systems (MEMS) devices,^[^ ^115^ ^]^ sensors,^[^ ^79^, ^151^ ^]^ surface functionalization,^[^ ^129^ ^]^ wearable electronics,^[^ ^152^ ^]^ and so forth. Further, due to its relative ease of operation and set‐up, EHD has the potential to be integrated into larger systems. For instance, EHD can be realistically combined into a system which also has inline metrology, as has been demonstrated by Pannier et al. who show that inline AFM can be incorporated.^[^ ^153^ ^]^ There exist opportunities to incorporate machine learning into the field of EHD printing. Early examples of this exist, such as the use of machine vision by Mieszczanek et al. to achieve real‐time process monitoring.^[^ ^154^ ^]^ This allowed them to demonstrate excellent process control in printing complex thick layer structures, and lends credence for the potential industrialization of EHD printing. Bioengineering applications too are likely to see a sharp increase in the near future.^[^ ^39^, ^89^, ^155^, ^156^ ^]^ With more work in tissue regeneration, soft matter printing, and biocompatible hydrogels, EHD will play a significant role in achieving new milestones.

Standardization of EHD printing is needed, especially since thus far, most such systems are built in a laboratory. Phung et al. make some recommendations for what parameters should be applied to achieve good printing both for drop on demand and near‐field continuous printing, however more consensus is required.^[^ ^93^ ^]^ Raje et al. recommend that a library of printing conditions be compiled for commercially available inks so that future EHD users may not have to spend excessive amounts of time optimizing printing conditions.^[^ ^157^ ^]^ One form of this standardization which already is widely used, is that of dimensionless numbers used to quantify the printing.^[^ ^13^, ^158^ ^]^

## 6 Conclusions

Having examined each of these parameters in more detail, it is possible to see just how much consideration is required to achieve the desired EHD printing outcome. EHD printing is a complex process. It can be made simpler by using Newtonian fluids with known properties including conductivity, surface tension, and viscosity. Knowing how these can affect the desired stable cone jet better informs the programming and selection of system parameters including flow rate, printing voltage, nozzle size, stand‐off height. Other factors such as ink dielectric strength, and environmental factors such as humidity and temperature can also affect the outcome, and so need to be controlled for reliable results. Certain ratios, such as *τ*_q_/*τ*_H_ can be helpful in determining whether printing can occur or not, and thus inform the choice of inks. Overall, success with EHD printing is achieved by doing the experiments. It is hoped that the discussion given here will assist users of EHD printing to understand the process a bit more and encourage more development.

## Conflict of Interest

The authors declare no conflict of interest.
